# Modeling hydrogen solubility in hydrocarbons using extreme gradient boosting and equations of state

**DOI:** 10.1038/s41598-021-97131-8

**Published:** 2021-09-09

**Authors:** Mohammad-Reza Mohammadi, Fahime Hadavimoghaddam, Maryam Pourmahdi, Saeid Atashrouz, Muhammad Tajammal Munir, Abdolhossein Hemmati-Sarapardeh, Amir H. Mosavi, Ahmad Mohaddespour

**Affiliations:** 1 Department of Petroleum Engineering, Shahid Bahonar University of Kerman, Kerman, Iran; 2 Gubkin National University of Oil and Gas, Moscow, 119991 Russia; 3 Department of Polymer Reaction Engineering, Faculty of Chemical Engineering, Tarbiat Modares University, Tehran, Iran; 4 Department of Chemical Engineering, Amirkabir University of Technology (Tehran Polytechnic), Tehran, Iran; 5 College of Engineering and Technology, American University of the Middle East, Kuwait, Kuwait; 6 Institute of Research and Development, Duy Tan University, Da Nang, 550000 Vietnam; 7 Faculty of Environment and Chemical Engineering, Duy Tan University, Da Nang, 550000 Vietnam; 8 Institute of Software Design and Development, Obuda University, Budapest, 1034 Hungary; 9 Department of Informatics, J. Selye University, 94501 Komarno, Slovakia

**Keywords:** Chemical engineering, Energy science and technology, Engineering

## Abstract

Due to industrial development, designing and optimal operation of processes in chemical and petroleum processing plants require accurate estimation of the hydrogen solubility in various hydrocarbons. Equations of state (EOSs) are limited in accurately predicting hydrogen solubility, especially at high-pressure or/and high-temperature conditions, which may lead to energy waste and a potential safety hazard in plants. In this paper, five robust machine learning models including extreme gradient boosting (XGBoost), adaptive boosting support vector regression (AdaBoost-SVR), gradient boosting with categorical features support (CatBoost), light gradient boosting machine (LightGBM), and multi-layer perceptron (MLP) optimized by Levenberg–Marquardt (LM) algorithm were implemented for estimating the hydrogen solubility in hydrocarbons. To this end, a databank including 919 experimental data points of hydrogen solubility in 26 various hydrocarbons was gathered from 48 different systems in a broad range of operating temperatures (213–623 K) and pressures (0.1–25.5 MPa). The hydrocarbons are from six different families including alkane, alkene, cycloalkane, aromatic, polycyclic aromatic, and terpene. The carbon number of hydrocarbons is ranging from 4 to 46 corresponding to a molecular weight range of 58.12–647.2 g/mol. Molecular weight, critical pressure, and critical temperature of solvents along with pressure and temperature operating conditions were selected as input parameters to the models. The XGBoost model best fits all the experimental solubility data with a root mean square error (RMSE) of 0.0007 and an average absolute percent relative error (AAPRE) of 1.81%. Also, the proposed models for estimating the solubility of hydrogen in hydrocarbons were compared with five EOSs including Soave–Redlich–Kwong (SRK), Peng–Robinson (PR), Redlich–Kwong (RK), Zudkevitch–Joffe (ZJ), and perturbed-chain statistical associating fluid theory (PC-SAFT). The XGBoost model introduced in this study is a promising model that can be applied as an efficient estimator for hydrogen solubility in various hydrocarbons and is capable of being utilized in the chemical and petroleum industries.

## Introduction

One of the fundamental properties for designing gas absorption and stripping columns in chemical industries is the solubility of gases in liquids^1^. While the basic principles of solubility thermodynamics are well known, it is still a challenging issue to accurately predict solubility for important industrial systems applying molecular thermodynamics alone. Nowadays, hydrogen is an eminent substance in the industry. Hydrogen plays a substantial role in industrial processes, hence the solubility of it in various hydrocarbon solutions such as fuels is very important for designing and optimal operating of these processes^2^. Hydrogen is a useful compound in the chemical and petroleum industries. The quality of heavy petroleum fractions can be upgraded through hydrovisbreaking or hydrocracking processes by adding hydrogen to them and increase the hydrogen to carbon ratio (H/C). The production of low sulfur fuels in the oil refining industry is such that large amounts of hydrogen are used for desulfurization plants^3–5^. Design and operating processes such as hydrogenation and hydrocracking, along with corresponding kinetic models, require hydrogen solubility data^6^. Pressure, temperature, and composition of solvents can remarkably affect the hydrogen solubility as a thermodynamic quantity. Increasing pressure and temperature have an increasing impact on the solubility of gases. Also, from the molar fraction point of view, as hydrocarbon carbon number increases, hydrogen solubility increases as demonstrated by experimental tests^2,7–9^. It is well known that traditional equations of state (EOSs) are limited in accurately predicting the solubility of hydrogen for the modeling of hydrogenation processes. There is a potential for energy waste and even a potential safety hazard in the hydrogenation process due to the overuse of hydrogen. Therefore, solubility data is very significant to predict the optimal amount of hydrogen in this process and can lead to improved plant safety. Performing experiments for heavy hydrocarbons due to the complexity of them is particularly difficult. Also, the risks associated with high-pressure or/and high-temperature conditions in industrial processes do not make extensive testing an attractive choice. Hence, modeling based on experimental data can be a good alternative.

The methods for predictions of hydrogen solubility in solvents such as hydrocarbons or petroleum mixture are mostly based on the application of empirical and semi-empirical models such as EOSs and are alike to those applied for solubility of other gases such as methane and CO_2_^10–15^. Shaw^16^ proposed a correlation for measuring the solubility of hydrogen in hydrocarbon solvents including heterocyclic, aromatic, and alicyclic type, by applying corresponding state theory^16^. Yuan et al.^17^ used molecular dynamics simulations to estimate the hydrogen solubility in heavy hydrocarbons for a range of pressures and temperatures. They concluded that a combination of the EOSs and molecular dynamics simulations can lead to more accurate and practical predictions for the hydrogen solubility at high pressures and temperatures^17^. Riazi and Roomi^5^ proposed a method for predicting the hydrogen solubility in hydrocarbons and their mixtures based on regular solution theory. Their procedure was based on calculating the parameter of hydrogen solubility according to the type of solvents or their molecular weight. The advantage of their method was that, unlike EOSs or other models, critical properties of solvent were not needed to calculate the hydrogen solubility. However, the need for other calculations in this method can still be considered a negative point^5^. Torres et al.^18^ applied the augmented Grayson Streed method^19^ to better model the solubility of hydrogen in heavy oil cuts. However, they noted that the homogeneous EOSs models could provide better results. The solubility of hydrogen in n-alcohols has been measured and modeled by d’Angelo and Francesconi^20^. Also, in their work, individual correlations as pseudo-Henry’s constants were used to better estimate hydrogen solubility^20^. Luo et al.^21^ experimentally investigated the hydrogen solubility in coal liquid and several hydrocarbons. They also proposed a mathematical model based on Henry’s law and the Pierotti method^21^. Yin and Tan^22^ obtained hydrogen solubility data in toluene in the presence of CO_2_ (i.e., ternary system H_2_ + toluene + CO_2_). An EOS named Peng–Robinson associated with the van der Waals mixing rule was used to model the vapor–liquid equilibrium (VLE) data^22^. Qian et al.^23^ used Peng–Robinson EOS to model a large dataset of various hydrogen-containing binary systems with the implementation of the group-contribution method for calculating temperature-dependent binary interaction parameters^23^. This method was previously been proposed by Jaubert and Mutelet to predict the VLE of hydrocarbons binary mixtures^24^. The solubility of hydrogen in several heavy normal alkanes has measured and modeled by Florusse et al.^2^. They used statistical associating fluid theory (SAFT) approach to model the hydrogen solubility after experiments. However, this method is a complex method due to the adjustable parameters and parameters required for any potential function^2^. Perturbed-Chain SAFT (PC-SAFT) EOS^25^ is another method that can be used to estimate the solubility of hydrogen in hydrocarbons. This method has been utilized to propose several models for prognostication of the solubility of hydrogen in hydrocarbons and heavy oils^6,26–28^. The classical EOSs, activity models, etc. require adjustable parameters, proper mixing rules, iterative calculations, etc. Traditional EOSs are only reliable in specific temperature and pressure ranges and have bounded flexibility for substances used.

Complex calculations in chemical and petroleum sciences have been facilitated by artificial intelligence (AI) methods in recent years. Regarding the use of artificial intelligence in the case of hydrogen solubility, Safamirzaei et al.^29^ have considered the hydrogen solubility in primary n-alcohols and after that, they applied artificial neural networks (ANNs) to overcome EOSs and simple correlations constraints in achieving best modeling^29^. Nasery et al.^30^ implemented Adaptive Neuro-Fuzzy Inference System (ANFIS) to estimate the solubility of hydrogen in heavy oil fractions^30^. Safamirzaei and Modarress^31^ modeled hydrogen solubility in heavy n-alkanes (C_46_H_94_, C_36_H_74_, C_28_H_58_, C_16_H_34_, and C_10_H_22_) by ANNs^31^. As can be seen in the literature studies, the issue of modeling hydrogen solubility in different solvents especially hydrocarbons has always been the focus of researchers. Also, according to the classification scheme of van Konynenburg and Scott^32^ and the updated version by Privat and Jaubert^33^, hydrogen-containing systems systematically show type III phase behavior, and such systems are acknowledged to be particularly difficult to correlate. Hence, there is a window for developing a more general model to estimate hydrogen solubility in hydrocarbons using AI methods, which accounts more influential variables, with higher precision. Due to the nature of data-driven soft computing techniques, such a comprehensive model can be developed by combining more data points and various operating conditions.

In the current work, we apply a total of 919 experimental hydrogen solubility data points for 26 different hydrocarbons accumulated at different operating conditions^1,2,8,11,14,21,34–44^. Advanced machine learning methods namely extreme gradient boosting (XGBoost), adaptive boosting support vector regression (AdaBoost-SVR), gradient boosting with categorical features support (CatBoost), light gradient boosting machine (LightGBM), and multi-layer perceptron (MLP) optimized by Levenberg–Marquardt (LM) algorithm are utilized to develop models for estimating the hydrogen solubility in hydrocarbons. Moreover, the validity of the proposed models is checked by applying statistical parameters and graphical error analyses. Also, several hydrogen solubility systems are estimated by the models developed in this work and five EOSs including Soave–Redlich–Kwong (SRK), Peng–Robinson (PR), Redlich–Kwong (RK), Zudkevitch–Joffe (ZJ), and perturbed-chain statistical associating fluid theory (PC-SAFT) to make a comparison between these models and EOSs.

## Data gathering

To accurately model hydrogen solubility in hydrocarbons, 919 experimental hydrogen solubility data were gathered from the literature^1,2,8,11,14,21,34–44^. Table 1 represents the sources of the experimental hydrogen solubility data used in this work along with the pressure range, temperature range, and uncertainty values for each system. Since the type of hydrocarbon dictates hydrogen solubility, a broad range of hydrocarbons was selected with properties represented in Table S1. Hydrocarbon families used in this study include alkane, alkene, cycloalkane, aromatic, polycyclic aromatic, and terpene.

**Table 1:** Hydrogen solubility database used for modeling in this work.

Fluid name	Temperature range (K)	Pressure range (MPa)	Hydrogen solubility (mole fraction in the liquid phase)	References
Butane	327.65–394.25 (± 0.05)	2.78–16.88 (± 0.005)	0.019–0.266 (± 0.04)	^43^
	297.05–388.75	2.25–10.72	0.021–0.111 (± 0.002)	^44^
Hexane	344.3–410.9 (± 0.1)	1.24–15.11 (± 0.007)	0.0105–0.143 (± 0.001)	^36^
	298.15–373.15 (± 0.1)	1.38–9.81 (± 0.002)	0.0107–0.0938 (± 0.02)	^39^
	213.15–298.15	0.101325	0.00037–0.00069 (± 1%)	^1^
Heptane	295	6.99–20.78 (± 0.5%)	0.0459–0.1289 (± 0.001)	^42^
Octane	295.15 (± 0.5)	0.68–1.38 (± 0.001)	0.00442–0.00801 (± 0.0002)	^38^
	298.15–373.15 (± 0.1)	2.4–15.27 (± 0.002)	0.0186–0.1371 (± 0.02)	^39^
	295	10.44–17.33 (± 0.5%)	0.066–0.1064 (± 0.001)	^42^
2,2,4-Trimethylpentane	295	6.99–20.78 (± 0.5%)	0.052–0.1452 (± 0.001)	^42^
Decane	283.17–449.63 (± 0.02)	1.23–14.21 (± 0.03%)	0.016–0.088 (± 0.001)	^2^
	344.3–423.2 (± 0.1)	3.71–17.39 (± 0.05)	0.0369–0.1288 (± 0.001)	^8^
	462.45–583.45 (± 0.2)	1.92–25.52 (± 0.03)	0.0251–0.5013 (± 0.001)	^37^
	293.15–373.15 (± 0.1)	2.04–10.35 (± 0.002)	0.0157–0.0884 (± 0.02)	^39^
Dodecane	344.3–410.9 (± 0.1)	1.42–13.24 (± 0.007)	0.0144–0.1252 (± 0.001)	^35^
Hexadecane	453.15–623.15	1.78–9.74	0.036–0.211 (± 0.001)	^21^
	298.13–448.17 (± 0.02)	1.15–15.13 (± 0.03%)	0.018–0.113 (± 0.001)	^2^
Eicosane	323.2–423.2 (± 0.1)	2.23–12.91 (± 0.05)	0.0273–0.1289 (± 0.001)	^8^
Octacosane	342.56–447.34 (± 0.02)	1.46–14.01 (± 0.03%)	0.031–0.178 (± 0.001)	^2^
	348.2–423.2 (± 0.1)	2.86–13.11 (± 0.05)	0.0452–0.1728 (± 0.001)	^8^
Hexatriacontane	357.53–447.43 (± 0.02)	1.37–14.34 (± 0.03%)	0.033–0.211 (± 0.001)	^2^
	373.2–423.2 (± 0.1)	3.56–16.75 (± 0.05)	0.0677–0.2271 (± 0.001)	^8^
Hexatetracontane	372.52–447.51 (± 0.02)	2.29–15.97 (± 0.03%)	0.065–0.257 (± 0.001)	^2^
1-Octene	295	6.99–20.78 (± 0.5%)	0.0435–0.1209 (± 0.001)	^42^
Benzene	303.15 (± 0.01)	2.02–4.60 (± 0.001)	0.0026–0.0126 (± 0.002)	^11^
	323.2–423.2 (± 0.1)	2.55–15.73 (± 0.06)	0.0103–0.0585 (± 0.001)	^14^
	295	6.99–17.33 (± 0.5%)	0.0172–0.0424 (± 0.001)	^42^
Toluene	303.15 (± 0.01)	1.22–4.41 (± 0.001)	0.0040–0.0145 (± 0.002)	^11^
	453.15–573.15	0.28–8.36	0.006–0.104 (± 0.001)	^21^
	298.15–373.15 (± 0.1)	0.874–10.12 (± 0.002)	0.0027–0.0471 (± 0.02)	^39^
	293–333 (± 0.1)	0.51–0.891 (± 0.0001)	0.00131–0.0034 (± 0.0001)	^40^
	295	6.99–17.33 (± 0.5%)	0.0216–0.0508 (± 0.001)	^42^
Ethylbenzene	295	10.44–17.33 (± 0.5%)	0.0332–0.0547 (± 0.001)	^42^
m-Xylene	295	10.44–17.33 (± 0.5%)	0.0343–0.056 (± 0.001)	^42^
Cumene	323 (± 0.2)	1.02–11.7 (± 0.035)	0.0041–0.0486 (± 0.003)	^41^
1,2,4-Trimethylbenzene	295	6.99–17.33 (± 0.5%)	0.0248–0.0571 (± 0.001)	^42^
Cyclohexane	303.15 (± 0.01)	0.88–4.74 (± 0.001)	0.0034–0.0196 (± 0.002)	^11^
	304–373	0.13–4.61	0.0006–0.0295 (± 0.0001)	^34^
	295	6.99–17.33 (± 0.5%)	0.0287–0.0683 (± 0.001)	^42^
Methylcyclohexane	303.15 (± 0.01)	1.23–4.32 (± 0.001)	0.0062–0.0218 (± 0.002)	^11^
	293–333 (± 0.1)	0.506–0.891 (± 0.0001)	0.00201–0.00479 (± 0.0001)	^40^
	295	6.99–20.78 (± 0.5%)	0.0332–0.0947 (± 0.001)	^42^
Naphthalene	373.2–423.2 (± 0.1)	4.29–19.39 (± 0.06)	0.0157–0.0567 (± 0.001)	^14^
	503.15–623.15	1.42–8.67	0.012–0.081 (± 0.001)	^21^
1,2,3,4-Tetrahydronaphthalene	453.15–623.15	1.53–9.19	0.014–0.085 (± 0.001)	^21^
Phenanthrene	383.2–423.2 (± 0.1)	5.89–21.69 (± 0.06)	0.0165–0.0557 (± 0.001)	^14^
Pyrene	433.2–423.2 (± 0.1)	5.17–19.73 (± 0.06)	0.0158–0.0575 (± 0.001)	^14^
Squalane	295.15 (± 0.5)	0.68–1.38 (± 0.001)	0.0062–0.01358 (± 0.0002)	^38^

To model hydrogen solubility in hydrocarbons, thermodynamic properties were considered for model development. In this work, molecular weight, critical pressure, and critical temperature of solvents along with pressure and temperature were selected as input parameters to the models. The hydrogen solubility (in terms of mole fraction) at different pressures and temperatures is set to be the model output. Moreover, a short statistical description of input and target parameters of the data bank applied for modeling is listed in Table 2. Using the uncertainty values of the experimental data in data-driven modeling can make the model really reliable. However, because uncertainty values (for test conditions and results of solubility tests) were not reported or fully reported in some papers, it was not possible to use them in modeling.

**Table 2:** Statistical information about the collected databank in this paper.

	Molecular weight (g/mol)	P_c_ (MPa)	T_c_ (K)	Pressure (MPa)	Temperature (K)	Mole fraction of hydrogen
Mean	200.06	6.28	661.23	6.85	378.68	0.07
Minimum	58.12	0.36	425.12	0.1013	213.15	0.00063
Maximum	647.2	41.08	938.2	25.52	623.15	0.5013
Median	142.28	2.11	617.7	6.14	373.15	0.0572
Mode	142.28	2.11	617.7	10.44	423.2	0.078
Kurtosis	1.38	4.48	– 0.76	0.81	1.75	6.43
Skewness	1.50	2.52	0.17	0.89	1.17	1.87

It is very important to apply different systems to achieve a comprehensive model for predicting hydrogen solubility in hydrocarbons. The characterization data for the 26 various hydrocarbons from 6 hydrocarbon families utilized for modeling are presented in Table S1. A databank including 919 data points was gathered from 48 different systems of the literature^1,2,8,11,14,21,34–44^, the statistical information of which is reported in Table 2. The carbon number of hydrocarbons is ranging from 4 to 46 corresponding to a molecular weight range of 58.12–647.2 g/mol. Also, the experimental hydrogen solubility data were collected in a broad range of operating temperatures, 213–623 (K) and pressures, 0.1–25.5 (MPa). According to the statistics reported in Table 2, the variation range and distribution of model input parameters are wide enough to provide a general model for estimating hydrogen solubility in hydrocarbons.

## Models implementation

### Extreme gradient boosting (XGBoost)

**Figure 1:**
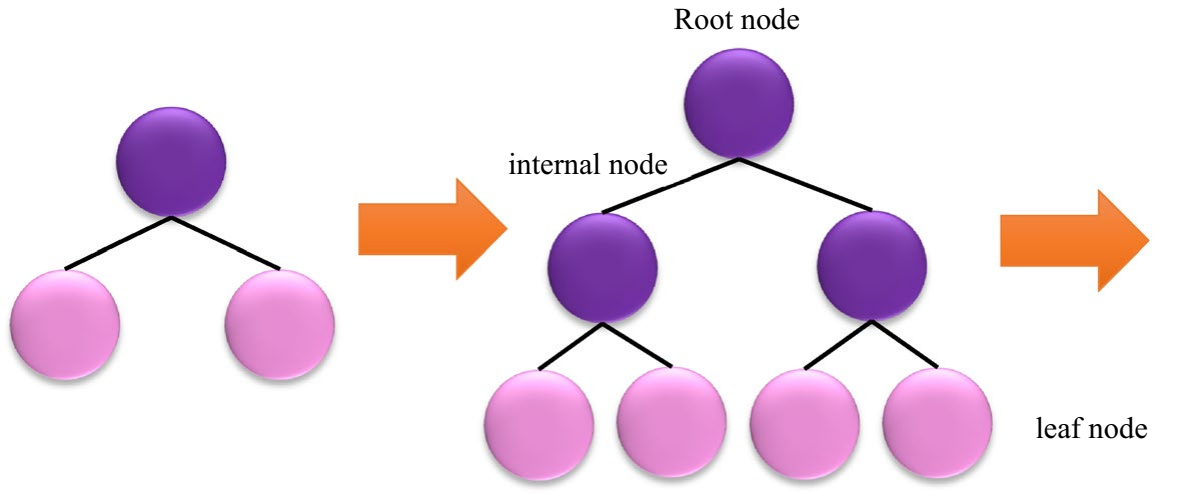
Level-wise tree growth in XGboost.

The main idea behind a tree-based ensemble technique is to utilize an ensemble of classification and regression trees (CARTs) such that the training data is fitted by the minimization of a regularized objective function. XGBoost is one of these tree-based models under the framework of gradient boosting decision tree (GBDT). To elaborate on the CART’s structure, every cart consists of (I) a root node, (II) internal nodes, and (III) leaf nodes as shown in Fig. 1. According to the binary decision practice, the root node which embodies the whole data set is subjected to be split into internal nodes, while the leaf nodes represent the ultimate classes. In order to build a robust ensemble in gradient boosting, a series of base CATRs are consecutively constructed where the weight of every individual CART needs to be tuned through the training process^45^.

To model the output *y* for a given dataset where *m* and *n* are dimension features and examples, respectively, an ensemble of *n* tress needs to be trained:

1 $$\begin{gathered} \hat{y}_{i} = \sum\limits_{{k = 1}}^{N} {f_{k} \left( {X_{i} } \right)} , \quad f_{k} \in f \hfill \\ With\; f = \left\{ {f(X) = \omega _{{q(x)}} } \right\},(q:\mathbb{R}^{m} \to T,\omega \;\in \mathbb{R}^{T} ) \hfill \\ \end{gathered}$$

where the example × is mapped by the decision rule *q(x)* to the binary leaf index. In Eqs. (1) and (2), *f* represents the space of regression trees, *f*_*k*_ is the kth independent tree, *T* denotes the number of leaves on the tree, and *ω* is the weight of the leaf.

The determination of the ensemble of trees is performed by the minimization of regularized objective function *L*:

2 $$\begin{gathered} L = \sum _{i}^{n} l(\hat{y}_{{_{i} }} ,y_{{_{i} }} ) + \sum _{k}^{N} \Omega (f_{k} ) \hfill \\ With\;\Omega (f) = \gamma T + \frac{1}{2}\lambda \left\| \omega \right\|^{2} \hfill \\ \end{gathered}$$

where *Ω* is the regularization term limiting the model intricacy, assisting the reduction of the overfitting; *l* denotes a differentiable convex loss function; *γ* stands for the minimum loss reduction which is needed to split a new leaf, and *λ* shows the regulation coefficient. It should be noted that *γ* and *λ* in these sets of equations help to soar the model variance and decrease the overfitting^46^.

In the gradient boosting approach, the objective function for every individual leaf is minimized through which more branched will be added iteratively.

3 $$L^{{(t)}} = \sum\limits_{{i = 1}}^{n} {\left\{ {l(y_{i} ,\hat{y}_{i} ^{{(t - 1)}} ) + f_{t} (X_{i} )} \right\}} + \Omega (f_{t} )$$

where *t* represents the *t*-th iteration in the aforementioned training process. To notably ameliorate the ensemble model, the XGBoost’s approach greedily adds the space of regression trees which is usually referred to as “greedy algorithm”. Therefore, the model output is iteratively updated through the minimization of the objective function:

4 $$\hat{y}_{i}^{{(t)}} = \hat{y}_{i}^{{(t - 1)}} + f_{t} (X_{i} )$$

The XGBoost benefits from the shrinkage strategy in which newly added weights are scaled after every step of boosting by a learning factor rate. This helps to diminish the effects of future new trees on every existing individual tree, thereby reducing the risk of overfitting^47^.

### Light gradient boosting machine (LightGBM)

**Figure 2:**
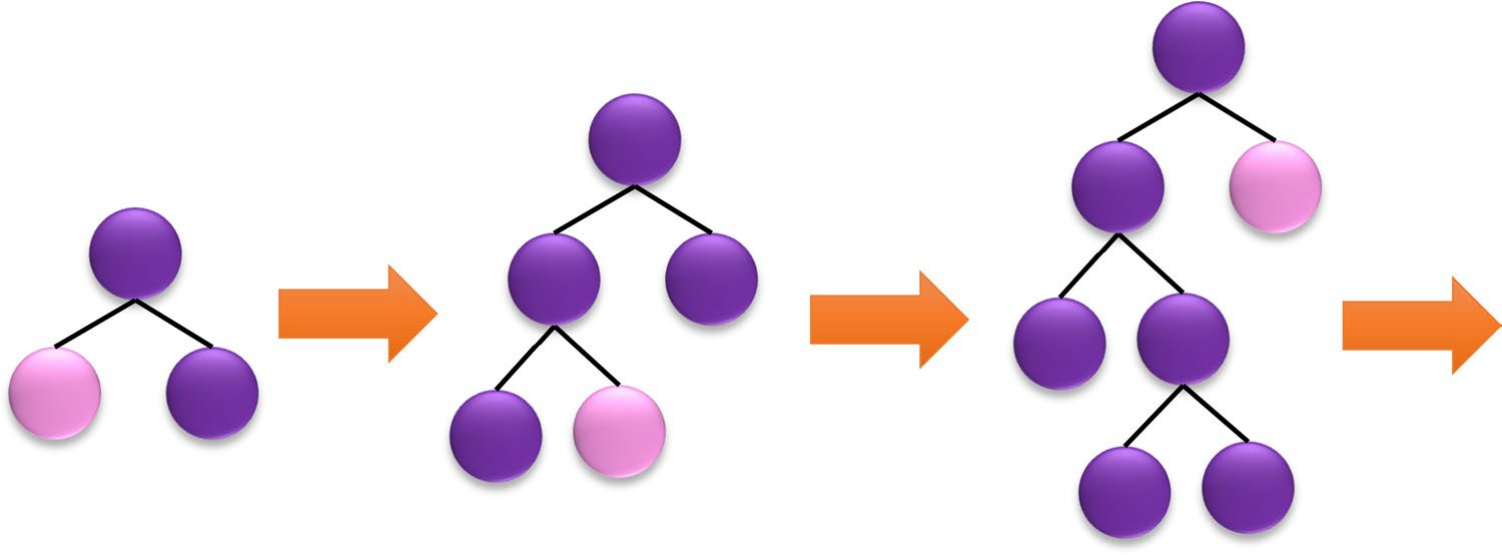
Leaf-wise tree growth in LightGBM.

Another new gradient learning framework built up upon the idea of the decision tree is LightGBM^48^. The salient features of LightGBM which dominates XGBoost are consuming less memory, utilizing a leaf-wise growth approach with depth restrictions, and benefiting from a histogram-based algorithm that expedites the training process^49^. Using the aforementioned histogram algorithm, LightGBM discretizes continuous floating-point eigenvalues into k bins, hence leading to building a k-width histogram. In addition, extra storage of pre-sorted results is not required in the histogram algorithm and values can be stored in an 8-bit integer after the feature discretization that reduces the memory consumption to 1/8. Nevertheless, this rough partitioning approach does decrease the model accuracy. LightGBM also uses a leaf-wise approach which is more effective than the traditional growth strategy named level-wise. The rationale behind this inefficiency in level-wise strategy is that the leaves from the same layer are considered at each step, thereby leading to a gratuitous memory allocation. Instead, the leaves with the highest branching gain are found at every step in the leaf-wise approach after which the algorithm continues to the branching cycle. Thus, the errors can be diminished and higher precision is achieved with the same number of segmentations compared to the horizontal direction. In Fig. 2, the strategy of leaf-wise tree growth is depicted. The downside of leaf orientation is growing deeper decision trees which unavoidably results in overfitting. However, LightGBM precludes this overfitting while furnishing high efficiency by applying a maximum depth limit to the leaf top^48,49^.

In the followings, calculations for LightGBM are shown^50^:

For a given training dataset $X = \left\{ {(x_{i} ,y_{i} )} \right\}_{{_{i = 1} }}^{m}$, LightGBM searches an approximation $\widehat{f}(x)$ to the function f*(x) to minimize the expected values of specific loss functions *L(y, f (x))*:

5 $$\mathop f\limits^{ \wedge } \left( x \right) = \arg \mathop {\min }\limits_{f} E_{y,x} L(y,f(x))$$

LightGBM ensembles many *T* regression trees $\sum_{t=1}^{T}{f}_{t }(x)$ to approximate the model. The regression trees are defined as *w*_*q(x)*_, $q \in \left\{ {1, \, 2,...,N} \right\}$, where *w* shows a vector representing the sample weights of leaf nodes, *N* stands for the number of tree leaves, and *q* represents the decision rule of trees. The model is trained in the additive form at step *t*:

6 $$G_{t} \cong \sum\limits_{i = 1}^{N} {L(y_{i} ,F_{t - 1} (x_{i} ) + f_{t} (x_{i} ))}$$

Newton's approach is used to approximate the objective function.

### Gradient boosting with categorical features support (CatBoost)

For categorical boosting, categorical columns are used in CatBoost which uses permutation techniques such as one_hot_max_size (OHMS) and target-based statistics. In this technique, a greedy method is used for each new split of the current tree which enables CatBoost to find the exponential growth of the feature combination^51^. The following steps are applied in CatBoost for every feature possessing more categories compared to OHMS:

1. Random subset formation of the records
2. Label conversion to integers
3. Categorical feature transformation to numeric, as follows:
  7 $$avgT\arg et = \frac{countInClass + prior}{{totalCount + 1}}$$

where *countInClass* counts targets with the value of one for a given categorical feature, and *totalCount* counts previous objects (the starting parameters determine the *prior* to count the objects)^52,53^.

### Adaptive boosting (AdaBoost)

For supervised classification, Freund and Schapire^54^ have suggested the AdaBoost system. In this model, reweighted data, that the eights are chosen reliability refers to the consistency of the output of the learners, are sequentially assumed in the week learners. This trick reduces the inexperienced learner in order to concentrate on the hard cases^55^. The following represent the key steps of the Adaboost technique:

- Defining Weights: $${w}_{j}=\frac{1}{n}, j=\mathrm{1,2},\dots .,n$$ ;
- For each *i*, set the training data to a weak learner ${Wl}_{i}(x)$ using weights and obtain the weighted error
  $${Err}_{i}=\frac{{\sum }_{j=1}^{n}{w}_{j}I({t}_{j}\ne {wl}_{i}\left(x\right))}{{\sum }_{j=1}^{n}{w}_{j}}, I\left(x\right)=\{\begin{array}{c}0 if x=false \\ 1 if x=true\end{array}$$
- For each *i*, determine weights for predictors as: ${\beta }_{i}=log\left(\frac{(1-{Err}_{i})}{{Err}_{i}}\right)$
- Modified data wights for each *i* to *N* ( *N* denotes the number of learners);
- Adjust weak learner for data test (*x*) as output.

In this study, support vector regressors (SVR) were applied as the weak learners in Adaboost systems.

### Support vector regression (SVR)

Support Vector machine (SVM) is a group of similar supervised machine learning algorithms that can be applied for both regression and clustering tasks^56^. SVR is a systematic technique for soft computation, with a well-established mathematical formulation. As it has been shown to be very stable for modeling multiple complex structures, this approach has gained significant interest. In the literature, the fundamental concept behind SVR is commonly presented^57^. Therefore, we present a short description of the SVR conception for the sake of brevity. SVR attempts to obtain a regression function *f(x)* for a given dataset $[\left({x}_{1}, {y}_{1}\right),\dots ..,\left({x}_{n}, {y}_{n}\right)]$ with $x\in {R}_{d}$ as the d-dimensional input space and $y\in R$ as the output vector dependent on the input data to estimate the output as below:

8 $$f(x) = w.\phi (x_{i} ) + b$$

where $b$ denotes bias vectors, $w$ shows the weight, and $\phi \left(x\right)$ refers to the function of the kernel. The following minimization problem proposed by Vapnik should be solved in order to achieve the right values of the weight and bias vectors^58^:

9 $$\begin{gathered} minimize\frac{1}{2}w^{T} w + C\sum\limits_{j = 1}^{N} {(\zeta_{j}^{ - } + \zeta_{j}^{ + } )} \hfill \\ \hfill \\ \left\{ \begin{gathered} (w.\phi (x_{i} ) + b) - y_{i} \le \varepsilon + \zeta_{j}^{ - } \hfill \\ y_{i} - (w.\phi (x_{i} ) + b) \le \varepsilon + \zeta_{j}^{ + } \hfill \\ \zeta_{j}^{ + } ,\zeta_{j}^{ - } \ge 0,i = 1,2,...,m \hfill \\ \end{gathered} \right. \hfill \\ \end{gathered}$$

where $T$ represents the transpose operator, $\varepsilon$ shows the error tolerance, *C* represents a positive regularization parameter that defines the variance from $\varepsilon$, ${\zeta }_{j}^{+}$ and ${\zeta }_{j}^{-}$ consider positive parameters, attempting to point out the lower and higher excess variations, respectively.

By means of the Lagrange multipliers, the previously discussed constrained optimization problem is taken into a dual function. This move then leads to the final solution, which is presented as follows:

10 $$f(x) = \sum\limits_{j = 1}^{n} {(a_{k} - a_{k}^{*} )K(x_{k} ,x_{l} ) + } b$$

where $K({x}_{k},{x}_{l})$ represents the kernel function; ${a}_{k}$ and ${a}_{k}^{*}$ represent the Lagrange multipliers that follow the *0 k* and *k C* constraints.

### Multilayer perceptron (MLP) neural network

**Figure 3:**
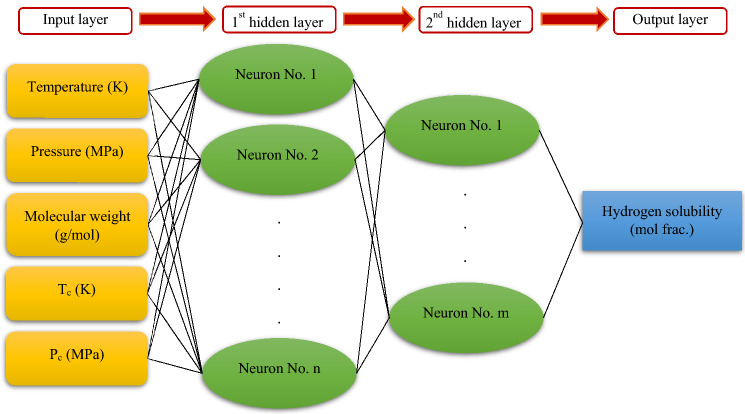
A schematic of the developed MLP neural network.

MLP is a class of feedforward ANNs that consists of various layers. The primary layer which is pertinent to the input data is the input layer, the last layer which corresponds to the output of the model is the output layer and the middle layers which process the information are hidden layers^59^. In the hidden layers, each neuron will connect to every neuron in the next and prior layers. The manner of calculating the value of every neuron in the output or hidden layers is as follows: the amount of every neuron in the prior layer which is multiplying in its corresponding particular weight is summed together and a bias factor is appended to these values. Then, the resulting value passes through an activation function^60^. Table S2 summarizes different activation functions along with their corresponding mathematical equations. The number of hidden layers and neurons in any hidden layer should be optimized to acquire a highly efficient and accurate model, usually using the empirical method. The performance of the MLP model depends on the optimization algorithms such as Levenberg–Marquardt (LM)^61^ applied to train this intelligent model. In this work, the MLP model which is developed on the basis of the LM optimization algorithm is dubbed MLP-LM . Figure 3 represents a schematic of the developed MLP in this work.

### The procedure of model development

For developing each model and take care of overfitting, we used grid search for optimizing hyperparameters of models. The hyperparameters used in grid search for each model were different, the importance of the hyperparameters was based on theoretical and practical aspects. The following hyperparameters were used for each model:

- For XGBoost: max_depth, n_estimators, learning_rate, min_child_weight, base_score.
- For LightGBM: Boosting type, objective, metric, learning rate, feature fraction, bagging fraction.
- For AdaBoost-SVR: learning rate, loss, epsilon, n_estimators, γ, C.
- For CatBoost: n_estimators, max_depth, learning rate.
- For MLP-LM: learning rate, Epoches.

The empirical method is also applied to determine the optimal number of hidden layers and neurons in any hidden layer for the MLP neural network.

In this work, we used k-fold cross-validation on our train dataset because it cares that every observation from the dataset has the chance of appearing in training and validation. For all models, we did use KFold 6 (as we know Kfold should not be too small or too high, and it depends on data size) so the value is picked up based on our data. It means we split the train data randomly into 6 folds and then fit the model using K-1 (which is 5 folds) and validate the model using the remaining fold.

### Equations of state (EOSs)

The analytical description of the relationship between volume, temperature, and pressure of a substance can be expressed by an EOS. The vapor–liquid–equilibria (VLE), volumetric behavior, and thermal properties of mixtures and pure substances can be described by this expression. The phase behavior of petroleum fluids is widely predicted by EOSs. As already mentioned, traditional EOSs offer poor predictions for the solubility of gases in solvents, especially in complex operating conditions. In this study, four cubic EOSs including SRK, PR, RK, and ZJ along with PC-SAFT as a type of SAFT EOSs are implemented to measure the hydrogen solubility in hydrocarbons and their precision in estimating the hydrogen solubility is compared with the proposed machine learning models. Conventional van der Waals one-fluid mixing rules are utilized in cubic EOSs. Table S3 shows the PVT relationships of the cubic EOSs and PC-SAFT equation in terms of the residual Helmholtz free energy. Furthermore, the parameters and mixing rules for the EOSs are presented in Table S4. Also, the pure-component PC-SAFT parameters for the substances used in this work are reported in Table S5. The binary interaction parameter (*k*_*ij*_) in van der Waals mixing rules characterizing molecular interactions between molecules of two components, can be a key parameter in estimating the solubility of a solute in a solvent in cubic EOSs. A similar *k*_*ij*_ parameter is introduced by applying the van der Waals one-fluid mixing rules to the perturbation terms in PC-SAFT EOS that corrects the segment-segment interactions of unlike chains. The optimized values of *k*_*ij*_ parameter for all EOSs in different hydrogen solubility systems are reported in Table S6.

## Model assessment

### Statistical error analysis

The following definitions have been implemented for the statistical parameters of standard deviation (SD), average absolute percent relative error (AAPRE), root mean square error (RMSE), coefficient of determination (*R*^2^), and average percent relative error (APRE) to assess the validation and accuracy of the models:

11 $$SD = \sqrt {\frac{1}{N - 1}\sum\limits_{i = 1}^{N} {\left( {\frac{{HS_{i,e} - HS_{i,p} }}{{HS_{{i,{\text{e}} }} }}} \right)}^{2} }$$

12 $$RMSE = \sqrt {\frac{1}{N}\sum\limits_{i = 1}^{N} {\left( {HS_{{i,{\text{e}} }} - HS_{i,p} } \right)}^{2} }$$

13 $$AAPRE = \frac{100}{N}\sum\limits_{i = 1}^{N} {\left| {\frac{{HS_{{i,{\text{e}} }} - HS_{i,p} }}{{HS_{{i,{\text{e}} }} }}} \right|}$$

14 $$APRE = \frac{100}{N}\sum\limits_{i = 1}^{N} {\left( {\frac{{HS_{{i,{\text{e}} }} - HS_{i,p} }}{{HS_{{i,{\text{e}} }} }}} \right)}$$

15 $$R^{2} = 1 - \frac{{\sum\limits_{i = 1}^{N} {(HS_{{i,{\text{e}} }} - HS_{i,p} )^{2} } }}{{\sum\limits_{i = 1}^{N} {(HS_{i,p} - \overline{{HS_{{i,{\text{e}} }} }} )^{2} } }}$$

In these formulas, *HS*_*i,e*_, *HS*_*i,p*_, and *N,* respectively, represent the experimental hydrogen solubility data and predicted values of hydrogen solubility in hydrocarbons by developed models, and the number of data points. The coefficient of determination which is represented almost everywhere by the *R*^2^ is one of the most well-known criteria for the goodness of fit of a model. *R*^2^ is an important statistical parameter that shows how well the model output corresponds to the experimental data and how valid the model is. If the *R*^2^ value is closer to 1, the fit of the model response to the experimental values is greater. The data scattering around zero deviation is assessed by RMSE. APRE and AAPRE measure the relative deviation and the relative absolute deviation from the target data, respectively. The measure of scattering is assessed by SD, which less value of it demonstrates a lower grade of dispersion.

### Graphical error analysis

Besides the statistical error analysis that has already been mentioned, visual graphical analysis can also help to understand the validity of the models developed in this work. The significant items are classified as follows:

Crossplot: in this graph, the estimated values of a model are plotted versus experimental values. If the finest fit line of the model estimation has no deviation from the 45° line and the computed data are mostly concentrated nearest to the unit slope line (Y = X), the performance of the model is excellent.

Error distribution plot: the presence or absence of error trend is checked by measuring the error scattering around the zero-error line. Here, the relative error (*E*_*i*_) is calculated through Eq. (16):

16 $${\text{E}}_{{\text{i}}} = \left[ {\frac{{HS_{{i,{\text{e}} }} - HS_{i,p} }}{{HS_{{i,{\text{e}} }} }}} \right] \times100 {\text{i }} = 1,2,3,...,n$$

Cumulative frequency graph: the cumulative frequency of data is sketched versus absolute relative error (*E*_*a*_). The higher cumulative frequency curve reveals that most of the estimations fall within the usual error range. In other words, the closer the curve to the vertical axis, the model error in estimating the high percentage of data is less. In this work, the *E*_*a*_ is calculated through Eq. (17):

17 $${\text{E}}_{a} = \left| {\frac{{HS_{{i,{\text{e}} }} - HS_{i,p} }}{{HS_{{i,{\text{e}} }} }}} \right| \times 100{\text{i }} = 1,2,3,...,n$$

Group error diagram: the data are divided into diverse ranges and their error at each range is calculated and sketched.

Trend plot: in this diagram, both target data and estimated values by the proposed model are sketched against the index of data points and their coverage and trend are tracked.

## Results and discussion

### Description of model development

The optimal values of the important hyperparameters along with the search interval of the hyperparameters tuned for the machine learning models implemented in this work are presented in Table 3.

**Table 3:** Optimal features for implemented models.

Model	Hyperparametr	Search range	Optimum value/feature
AdaBoost−SVR	γ	0.1–0.0001	0.005
	Epsilon	0.1–0.0001	0.0001
	C	1–500	100
	Learning rate	0.01–0.9	0.35
	loss	–	Exponentioal
	Max_depth	1–14	8
MLP-LM	Train function	–	LM
	Hidden layer (s)	1–20	[16 * 8]
	Activation function	[sigmoid-ReLU]	ReLU
	Epoches	100–300	250
	Learning rate	0.001–0.1	0.095
LightGBM	n_estimators	1–2000	800
	Learing_rate	0.01–0.9	0.29
	Max_depth	1–14	12
	metric	Rmse, mse, mape	Mape
	Feature_fraction	0.4–1	0.9
	bagging fraction	0.6–0.9	0.8
XGBoost	n_estimators	1–2000	1700
	Learing_rate	0.01–0.9	0.09
	Subsample	0.1–1	0.6
	Max_depth	1–16	15
	Feature_fraction	0.4–0.95	0.8
	Base score	0.25–1	1
	min_child_weight	1–4	2
CatBoost	n_estimators	1–2000	100
	Learing_rate	0.01–0.9	0.3
	Subsample	0.1–1	0.8
	Max_depth	1–16	15

In Table 3, n_estimators show the number of trees; subsample is subsample ratio of the training instance; C denotes a degree of importance that is given to misclassifications; max_depth represents maximum depth of a tree; min_child_weight is the minimum sum of instance weight (hessian) needed in a child; bagging_fraction shows the fraction of data to be used for each iteration; feature_fraction is parameters randomly selected in each iteration for building trees; learning_rate controls the impact of each tree on the final outcome; base_score represents the initial prediction score of all instances; epsilon is a parameter affect the number of support vectors applied to construct the regression function; γ shows kernel coefficient, and epochs show the number of times that the learning algorithm is passed through a full training dataset.

### Statistical assessment of the developed models

To identify the most accurate model, we should compare the developed models using statistical factors including, *R*^2^, AAPRE (%), SD, APRE (%), and RMSE. The calculated values for these parameters are reported in Table 4. The results reveal that among all developed models, XGBoost provides the most accurate predictions, followed by AdaBoost-SVR, LightGBM, CatBoost, and MLP−LM models, respectively. Based on Table 4, AAPRE values of 2.14% for the testing set, 1.71% for the training set, and 1.81% for the total set of data, suggest that the XGBoost model has the most accurate estimation of hydrogen solubility in hydrocarbons. However, Table 4 reveals that other models also display good accuracy.

**Table 4:** Statistical error analysis for the models developed in this work.

Statistical factors	RMSE	APRE %	AAPRE %	SD	R^2^
**XGBoost**					
Train	0.0006	0.009	1.707	0.043	0.9999
Test	0.0007	0.128	2.145	0.065	0.9998
Total	0.0007	0.039	1.815	0.048	0.9998
**CatBoost**					
Train	0.0014	0.194	4.678	0.201	0.9994
Test	0.0016	− 1.551	4.808	0.161	0.9990
Total	0.0015	− 0.161	4.705	0.193	0.9993
**LightGBM**					
Train	0.0047	− 0.980	3.374	0.133	0.9938
Test	0.0038	− 1.422	4.087	0.137	0.9946
Total	0.0045	− 1.073	3.517	0.134	0.9940
**AdaBoost-SVR**					
Train	0.0011	− 2.559	3.256	0.142	0.9996
Test	0.0014	− 3.004	3.960	0.125	0.9928
Total	0.0012	− 2.651	3.401	0.139	0.9995
**MLP−LM**					
Train	0.0054	− 1.859	5.809	0.087	0.9918
Test	0.0049	− 2.694	6.786	0.114	0.9908
Total	0.0053	− 2.042	6.011	0.093	0.9917

For a comparative evaluation of the models developed in this work with five EOSs, 30 hydrogen solubility data points in three different systems including hydrocarbons with low, medium, and high molecular weight collected from the literature^8,11,39^ were estimated by these models. Predictions of models along with the results calculated by the EOSs are presented in Table 5. The AAPRE reported in Table 5 is much higher for the EOSs than the machine learning models. ZJ EOS with an AAPRE of 15.78% has the best calculations for hydrogen solubility in hydrocarbons among the other cubic EOSs. Also, PC-SAFT as a modern type of EOSs shows good estimates with AAPRE of 9.56% and has superior performance compared to traditional cubic EOSs. All machine learning models have good predictions and show a significant advantage over EOSs. XGBoost model has the best performance among all models and EOSs with an AAPRE of 1.92%. It is noteworthy that uncertainty values are different for different systems. According to our studies, AAPRE values reported in Table 5 can vary about 5–10% due to uncertainty values, but it is better to trust the reported experimental values in the literature.

**Table 5:** Comparison of proposed models’ performance in this work with EOSs.

Hydrocarbon	Data no.	Hydrogen solubility, mol frac										
		Experimental	XGBoost	CatBoost	LightGBM	AdaBoost-SVR	MLP-LM	PR	SRK	RK	ZJ	PC-SAFT
Benzene	1	0.00267	0.00243	0.00368	0.00303	0.00395	0.00315	0.0035	0.0031	0.0034	0.0029	0.00232
	2	0.00395	0.00359	0.00386	0.00325	0.00430	0.00442	0.0041	0.0036	0.0039	0.0036	0.00269
	3	0.00468	0.00454	0.00428	0.00469	0.00601	0.00571	0.0061	0.0054	0.0057	0.0038	0.00397
	4	0.00560	0.00552	0.00675	0.00561	0.00719	0.00671	0.0074	0.0066	0.0069	0.0051	0.00477
	5	0.00601	0.00604	0.00571	0.00600	0.00794	0.00778	0.0081	0.0072	0.0075	0.0046	0.00521
	6	0.00761	0.00783	0.00737	0.00842	0.00956	0.00975	0.01	0.0089	0.0093	0.0075	0.00647
	7	0.00983	0.01037	0.00859	0.00983	0.01140	0.01164	0.013	0.0115	0.012	0.008	0.00834
	8	0.01077	0.01136	0.00918	0.01078	0.01308	0.01272	0.0143	0.0127	0.0132	0.0091	0.00918
	9	0.01268	0.01253	0.01436	0.01268	0.01683	0.01510	0.0168	0.0149	0.0155	0.0109	0.01079
Octane	10	0.01961	0.01857	0.01681	0.02047	0.01961	0.01985	0.0227	0.022	0.0275	0.0156	0.01692
	11	0.04472	0.04406	0.04451	0.04473	0.04472	0.04712	0.052	0.0503	0.0625	0.0359	0.03943
	12	0.06806	0.06815	0.06738	0.07083	0.06802	0.07290	0.0786	0.0759	0.0937	0.0544	0.06056
	13	0.09073	0.09003	0.09243	0.09073	0.08871	0.09755	0.1026	0.0988	0.1213	0.0711	0.08013
	14	0.01861	0.01860	0.01855	0.01861	0.01904	0.01814	0.0207	0.0205	0.0257	0.0157	0.01671
	15	0.04841	0.04800	0.04651	0.04534	0.04841	0.04856	0.0527	0.0519	0.0651	0.04	0.04313
	16	0.076	0.07544	0.07551	0.07719	0.07408	0.07553	0.0823	0.0808	0.1007	0.0626	0.06859
	17	0.1022	0.10177	0.10019	0.10220	0.10220	0.10079	0.1089	0.1066	0.1321	0.083	0.09241
	18	0.0286	0.02825	0.02873	0.02860	0.03000	0.02969	0.0334	0.0332	0.0412	0.0274	0.02801
	19	0.06582	0.06700	0.06595	0.05796	0.06711	0.06581	0.0742	0.0732	0.0917	0.0608	0.06294
	20	0.10491	0.10402	0.10265	0.10492	0.10373	0.11202	0.1163	0.1139	0.1419	0.0951	0.10011
	21	0.13701	0.13738	0.13683	0.13698	0.13701	0.13579	0.1483	0.1445	0.1791	0.1212	0.12931
Octacosane	22	0.0503	0.05084	0.05200	0.05234	0.05240	0.05159	0.0619	0.0652	0.1008	0.0403	0.04883
	23	0.0524	0.05296	0.05251	0.05238	0.05240	0.05303	0.0637	0.0671	0.1042	0.0415	0.05028
	24	0.0747	0.07369	0.07386	0.07309	0.07470	0.07558	0.0913	0.0957	0.1523	0.0601	0.07222
	25	0.0921	0.09211	0.09479	0.09210	0.09155	0.09173	0.1119	0.1169	0.1862	0.0742	0.08871
	26	0.1047	0.10449	0.10370	0.10469	0.10470	0.10211	0.1265	0.1318	0.2093	0.0844	0.10042
	27	0.1235	0.12420	0.12469	0.12386	0.12350	0.12008	0.1482	0.154	0.2422	0.0997	0.11799
	28	0.1407	0.14109	0.14115	0.14070	0.14070	0.14074	0.1695	0.1755	0.2729	0.115	0.13526
	29	0.1511	0.15027	0.15209	0.14623	0.15110	0.14782	0.1823	0.1884	0.2907	0.1243	0.14565
	30	0.1728	0.17321	0.17476	0.17280	0.17280	0.17083	0.2087	0.2149	0.3258	0.1439	0.16763
AAPRE %		–	1.92	5.24	2.67	8.68	7.85	19.87	16.63	49.95	15.78	9.56

To further evaluate the validity and reliability of the XGBoost model, an external validation dataset containing 413 hydrogen solubility data in 18 different hydrocarbons, including 6 new hydrocarbons (i.e. ethane, propane, ethene, 1-hexene, 1-heptene, and diphenylmethane) over a wide range of operating temperatures (98–701 K) and pressures (1.03–78.45 MPa), were collected from the literature. The properties of all hydrocarbons used in this work are presented in Table S1. Table 6 describes this validation dataset of hydrogen solubility data. This dataset is completely outside the training and testing sets used for modeling in this paper. Hence, it allows evaluating the performance of the model outside the modeling data sets. AAPRE values for each system are calculated using experimental data and predictions of the XGBoost model. The AAPRE values reported in Table 6 show that the XGBoost model has good predictions for all systems, even for new hydrocarbons not used in modeling. Overall AAPRE of 1.78% for this validation dataset shows the high validity of the XGBoost model in predicting hydrogen solubility in hydrocarbons.

**Table 6:** Validation dataset for evaluation of XGBoost model.

Fluid name	Temperature range (K)	Pressure range (MPa)	Hydrogen solubility (mole fraction in the liquid phase)	No. of data	References	AAPRE % using XGBoost model
Ethane	148.15–223.15 (± 0.1)	2.03–8.11 (± 0.01)	0.0061–0.0557 (± 1%)	16	^62^	1.90
Propane	98.15–148.15 (± 0.05)	1.03–20.68 (± 0.02)	0.0021–0.0473 (± 2%)	23	^63^	5.11
Butane	144.26–244.26 (± 0.5)	2.07–51.36 (± 0.13)	0.008–0.229 (± 0.0025)	26	^64^	2.28
Hexane	308.35 (± 0.1)	5.1–15.17 (± 0.13)	0.0328–0.0908 (± 0.002)	8	^65^	0.69
Heptane	424.15–498.85 (± 0.1)	2.45–78.45 (± 0.01)	0.02–0.71 (± 3%)	26	^66^	0.41
Decane	358.15–483.15 (± 1)	4.05–30.4 (± 0.05)	0.036–0.345 (± 3%)	12	^67^	0.39
	503 (± 0.5)	1.48–10.1 (± 0.03)	0.0178–0.1507 (± 0.6%)	6	^68^	2.17
Dodecane	366.5–422 (± 0.2)	3.62–34.72 (± 1%)	0.0373–0.299 (± 1%)	11	^69^	1.05
Hexadecane	461.65–622.85 (± 0.1)	2.009–25.27 (± 0.01)	0.0311–0.4458 (± 1%)	21	^70^	0.59
Cyclohexane	310.9–407.6 (± 0.02)	3.45–62.05 (± 0.003)	0.0135–0.2644 (± 0.001)	46	^71^	0.82
Toluene	461.83–575.15 (± 0.1)	2.02–25.37 (± 0.05%)	0.0082–0.3935 (± 0.001)	25	^72^	2.22
Benzene	433.15–533.15 (± 0.1)	1.9–17.803 (± 0.05%)	0.0071–0.1317 (± 0.001)	49	^73^	2.28
	288.15 (± 0.1)	5.01–49.3 (± 0.35%)	0.0114–0.102 (± 0.5%)	11	^74^	1.52
1-Hexene	333.15–443.15 (± 1)	4.05–30.4 (± 0.05)	0.04–0.38 (± 3%)	12	^67^	0.34
1-Heptene	333.15–473.15 (± 1)	4.05–30.4 (± 0.05)	0.028–0.353 (± 3%)	12	^67^	0.56
1-Octene	328.15–463.15 (± 1)	4.05–30.4 (± 0.05)	0.024–0.318 (± 3%)	12	^67^	1.18
Phenanthrene	398.2–473.2	2.613–25.23	0.0094–0.0840 (± 0.001)	24	^75^	1.44
Diphenylmethane	462.75–701.65 (± 0.7)	2.026–25.33 (± 0.03)	0.0123–0.3056 (± 1%)	27	^76^	0.82
Ethene	123.15–248.15 (± 0.1)	2.03–8.11 (± 0.01)	0.0053–0.0603 (± 1%)	22	^62^	6.30
1,2,3,4-Tetrahydronaphthalene	462.75–662.25 (± 0.05)	2.03–25.33 (± 0.1)	0.0118–0.2824 (± 1%)	24	^77^	1.16
Overall	98.15–701.65	1.03–78.45	0.0021–0.71	413	-	1.78

### Visual error analysis

**Figure 4:**
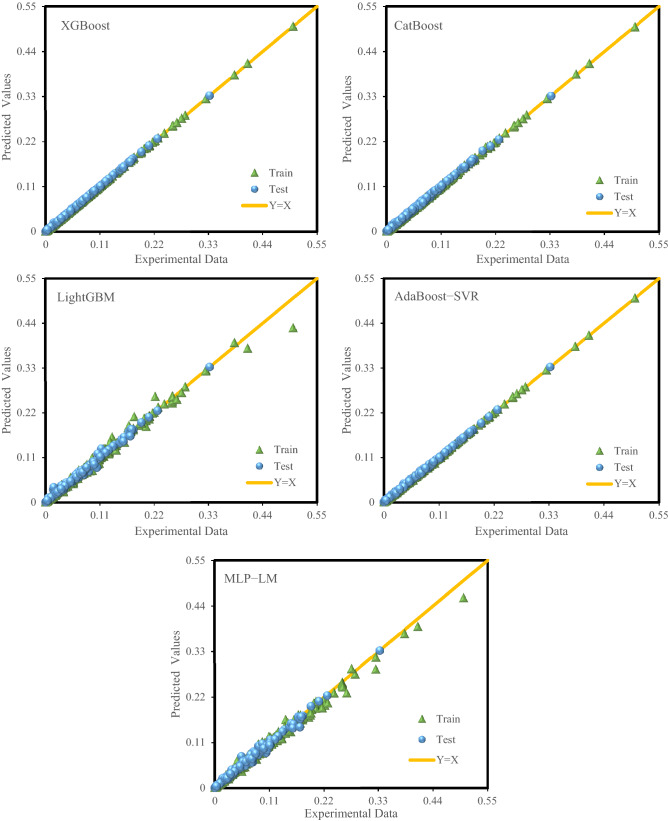
Crossplot of prediction of hydrogen solubility in hydrocarbons by the models versus experimental data.

**Figure 5:**
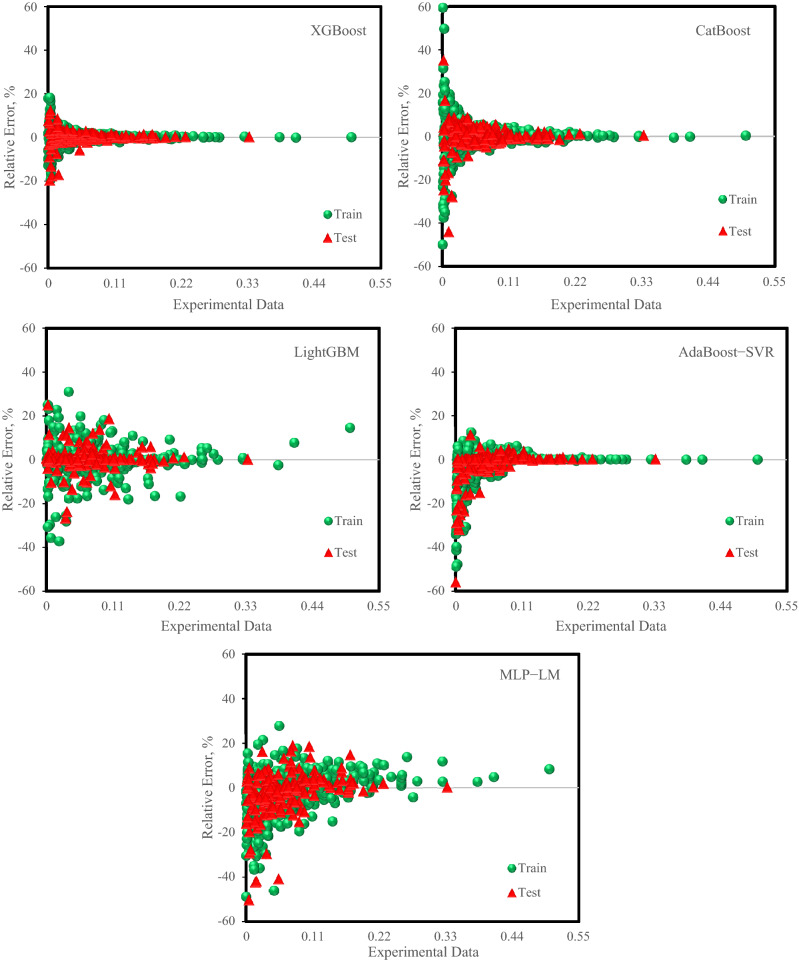
Error distribution graphs of the proposed models for test and training sets.

For a more detailed assessment of the accuracy of the proposed models, visual analysis applying the crossplot of predicted hydrogen solubility against the corresponding experimental values was depicted in Fig. 4. Besides, Fig. 5 presented the error distribution diagram for each of the two testing and training sets of all models. Figure 4 demonstrates that the high concentration of data points surrounding the 45° line for all models. However, the XGBoost model performs much better than other models, indicating its high reliability for predicting hydrogen solubility in hydrocarbons. The relative errors among experimental hydrogen solubility and estimated values by the proposed models versus the experimental data for the test and training sets are illustrated in Fig. 5. This figure demonstrates that the relative errors of XGBoost and AdaBoost-SVR models are highly near the zero-error line, but the errors of the predictions of CatBoost, LightGBM, and MLP-LM models are not as low as the XGBoost and AdaBoost-SVR models. The maximum percent relative error among the estimated hydrogen solubility values and the experimental data for the XGBoost model is 19%. Figures 4 and 5 reflect the significant extent of agreement between the experimental hydrogen solubility data and the XGBoost model predictions.

Figure S1 represents the trend plot of the predicted values of hydrogen solubility in hydrocarbons for all proposed models and the experimental hydrogen solubility data versus the index of data points. As demonstrated in Fig. S1 in the Supplementary file, all models show good overlap between the estimated hydrogen solubility data and the experimental values, but the degree of overlap is excellent for the XGBoost model.

Figure S2 depicts the cumulative frequency of the data versus *E*_*a*_ for all developed models. Based on this figure, more than 70% of estimated hydrogen solubility by the XGBoost model have an absolute relative error < 1.3%, as well as more than 90% of the estimated data, have an absolute relative error < 3.6%. However, for the AdaBoost-SVR, LightGBM, CatBoost, and MLP-LM models respectively 81%, 79%, 73%, and 48% of predicted hydrogen solubility data have an absolute relative error < 3.6%, indicating the high validity of the XGBoost model.

**Figure 6:**
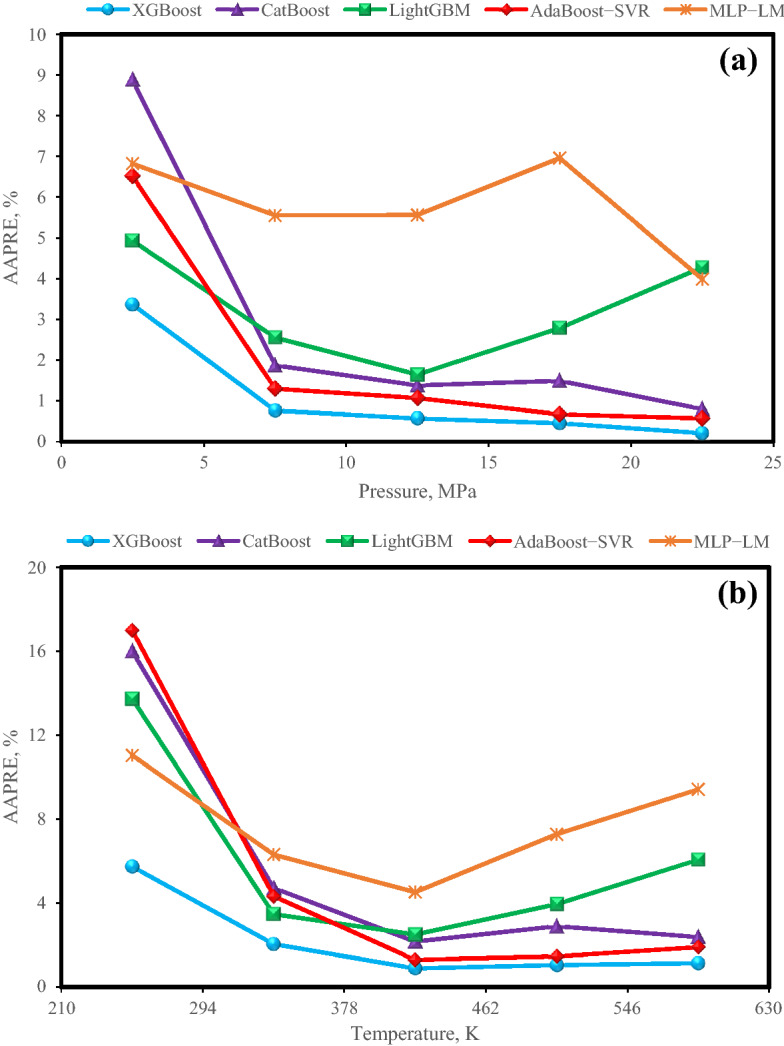
Graph of Group error for all models for various ranges of (**a**) pressure and (**b**) temperature.

Operating pressure and temperature greatly affect the solubility of hydrogen in hydrocarbons. As mentioned earlier, predicting hydrogen solubility under high-pressure/ igh-temperature conditions in various industries, is very important and the safety and efficiency of industrial processes depend on it. Figure 6 presents the validity of models at selected values of pressure and temperature ranges by applying the group error plots. It is worth noting that the group error analysis is performed by splitting all data into various ranges of pressure (i.e. 0–5 MPa, 5–10 MPa, 10–15 MPa, 15–20 MPa, and 20–25 MPa) and temperature (i.e. 210–294 K, 294–378 K, 378–462 K, 462–546 K, and 546–630 K) to investigate the validity of the proposed models at various ranges of these important parameters. AAPRE was calculated for the mentioned intervals and plotted in Fig. 6a for pressure parameter and Fig. 6b for temperature parameter. As can be seen in Fig. 6, LightGBM and MLP-LM models have relatively higher errors in low and high pressures and temperatures. Also, CatBoost and AdaBoost-SVR models have relatively higher errors in low pressures and temperatures. XGBoost model has the lowest error among all models for different temperature and pressure operating conditions, which proves the previous claims of good performance of this model.

### Trend analysis

**Figure 7:**
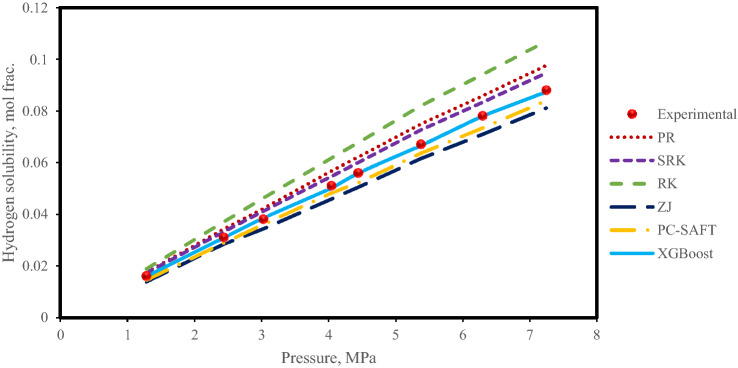
Estimated hydrogen solubility in n-Decane at a high-temperature of 432 K.

At the next stage, several different analyses were performed to assess the performance of the XGBoost model in different systems of hydrogen solubility in hydrocarbons. First, the impact of pressure on the hydrogen solubility in n-Decane at a high temperature of 432 K^2^ is evaluated in Fig. 7. The hydrogen solubility values predicted by the XGBoost model for this system along with the values calculated by the EOSs are demonstrated in Fig. 7. As indicated in this figure, at high-temperature conditions, the deviation between traditional RK EOS calculations and experimental data is high, but the other EOSs and XGBoost model predict experimental data excellently. As expected, the solubility of hydrogen in the n-Decane increases with increasing pressure. However, cubic EOSs slightly overestimate or underestimate the increase in solubility with increasing pressure at high temperatures, while the XGBoost model follows the trend very well. PC-SAFT EOS also has good predictions with low deviation from experimental data and outperforms traditional cubic EOSs.

**Figure 8:**
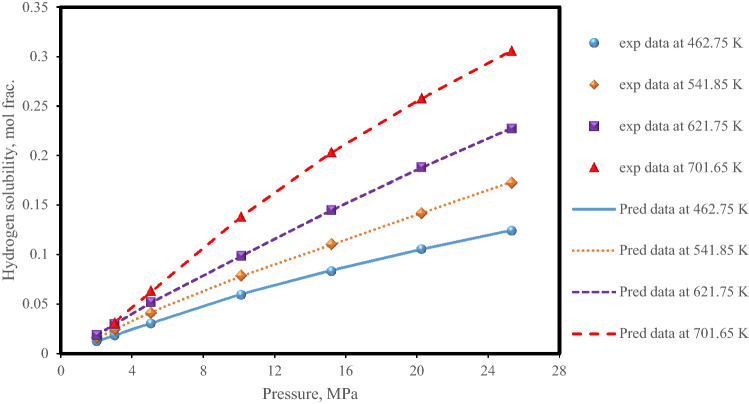
Experimental data with XGBoost model predictions of hydrogen solubility in diphenylmethane under different operating conditions.

Next, the hydrogen solubility data in a hydrocarbon named diphenylmethane^76^ with a molecular weight of 168.23 and a carbon number of 13 are predicted by the XGBoost model at high temperature and pressure conditions (Fig. 8). Again, as depicted in Fig. 8, the XGBoost model correctly detects data trends and provides excellent forecasts. As can be seen, the effect of temperature increase along with increasing pressure on hydrogen solubility is correctly predicted by the XGBoost model.

**Figure 9:**
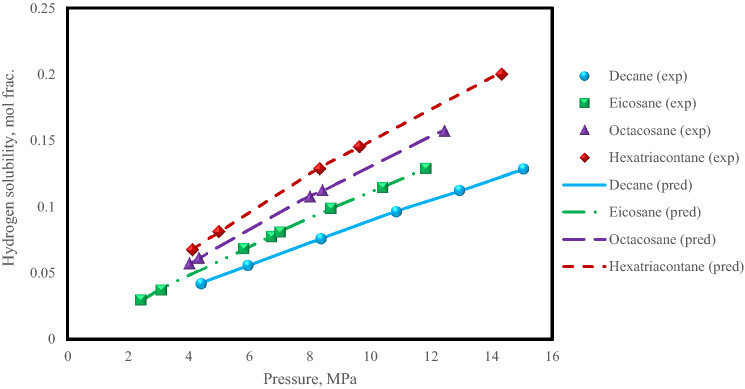
The solubility of hydrogen in several hydrocarbons with different carbon numbers for the XGBoost model with experimental data.

As mentioned earlier, the solubility of hydrogen increases with an increasing carbon number of hydrocarbons^2,7–9^. Therefore, the predictions of the XGBoost model for the solubility of hydrogen in several hydrocarbons with different carbon numbers (decane, eicosane, octacosane, and hexatriacontane) at a temperature of 373 K, which have been studied experimentally in literature^8^, are presented in Fig. 9. In this case, as well, the estimations of the XGBoost model are in good agreement with the reported experimental hydrogen solubility data for all these hydrocarbons.

## Conclusions

In this work, five robust machine learning models were introduced for estimating the hydrogen solubility in hydrocarbons as a function of critical pressure, critical temperature, and molecular weight of solvents along with pressure and temperature operating conditions. A databank including 919 data points gathered from 48 different systems of the 26 various hydrocarbons was applied to model the hydrogen solubility. Implementing the techniques of XGBoost, CatBoost, LightGBM, AdaBoost-SVR, and MLP-LM revealed that the estimations of hydrogen solubility in hydrocarbons from the five proposed models reached the AAPRE of 1.81%, 3.40%, 3.52%, 4.70%, and 6.01% for XGBoost, AdaBoost-SVR, LightGBM, CatBoost, and MLP-LM , respectively. XGBoost is introduced as the best-proposed model in this work based on graphical and statistical error analysis. Evaluation of the XGBoost model with an external validation dataset containing 413 hydrogen solubility data in 18 different hydrocarbons over a wide range of operating temperatures (98–701 K) and pressures (1.03–78.45 MPa) also proved the validity and reliability of the XGBoost model in predicting hydrogen solubility in hydrocarbons. Also, the calculation of hydrogen solubility in hydrocarbons for several different systems by EOSs showed that PC-SAFT has the best predictions for hydrogen solubility in hydrocarbons among the other EOSs. However, ZJ EOS also outperformed another cubic EOSs.

## Supplementary Information

Supplementary Information 1.

## Abbreviations

ZJ: Zudkevitch–Joffe EOS
XGBoost: EXtreme gradient boosting
VLE: Vapor–liquid equilibrium
SVR: Support vector regression
SD: Standard deviation
SVM: Support vector machine
SAFT: Statistical associating fluid theory
SRK: Soave–Redlich–Kwong EOS
ReLU: Rectified linear unit
RMSE: Root mean square error
RK: Redlich–Kwong EOS
pred: Predicted
PC-SAFT: Perturbed-chain statistical associating fluid theory
PR: Peng–Robinson EOS
OHMS: One_hot_max_size
MLP-LM: Multilayer perceptron optimized by Levenberg–Marquardt algorithm
LightGBM: Light gradient boosting machine
HS: Hydrogen solubility
exp: Experimental
EOSs: Equations of state
EOS: Equation of state
CARTs: Classification and regression trees
CatBoost: Gradient boosting with categorical features support
AAPRE: Average absolute percent relative error
ANFIS: Adaptive neuro-fuzzy inference system
APRE: Average percent relative error
AdaBoost-SVR: Adaptive boosting support vector regression

## Subscript and superscript

R^2^: Coefficient of determination
E_i_: Relative error
E_a_: Absolute relative error
