# Supplementary material for: Modeling hydrogen solubility in hydrocarbons using extreme gradient boosting and equations of state
Source: Sci Rep. 2021 Sep 9;11:17911. doi: 10.1038/s41598-021-97131-8 (PMC8429697; doi:10.1038/s41598-021-97131-8)
Supplement: Supplementary file 1 — Supplementary Information 1. [file 41598_2021_97131_MOESM1_ESM.docx]

**Modeling hydrogen solubility in hydrocarbons using extreme gradient boosting and equations of state**

Mohammad-Reza Mohammadi ^1^, Fahime Hadavimoghaddam ^2^, Maryam Pourmahdi ^3^, Saeid Atashrouz^4^, Muhammad Tajammal Munir ^5^, Abdolhossein Hemmati-Sarapardeh ^1, 6, 7,^ *, Amir Mosavi ^8, 9,^ *, Ahmad Mohaddespour ^5,^ *

*^1^ Department of Petroleum Engineering, Shahid Bahonar University of Kerman, Kerman, Iran*

*^2^ Gubkin National University of Oil and Gas, Moscow, 119991, Russia*

*^3^ Department of Polymer Reaction Engineering, Faculty of Chemical Engineering, Tarbiat Modares University, Tehran, Iran*

*^4^ Department of Chemical Engineering, Amirkabir University of Technology (Tehran Polytechnic), Tehran, Iran*

*^5^ College of Engineering and Technology, American University of the Middle East, Kuwait*

*^6^ Institute of Research and Development, Duy Tan University, Da Nang 550000, Vietnam*

*^7^ Faculty of Environment and Chemical Engineering, Duy Tan University, Da Nang 550000, Vietnam*

*^8^* *John von Neumann Faculty of Informatics, Obuda University, 1034 Budapest, Hungary*

**Supplementary file:**

**Fig. S1.** Estimated values of hydrogen solubility by the models and experimental values versus the data index.

**Fig. S2.** Graph of cumulative frequency against E_a_ for all models.

**Table S1.** The properties of the hydrocarbons used in the present study.

| P_c_ (MPa) | T_c_ (K) | Molecular weight (g/mol) | Carbon  Number | Hydrocarbon type | Fluid name |
| --- | --- | --- | --- | --- | --- |
| 4.872 | 305.32 | 30.07 | 2 | Alkane | Ethane |
| 4.248 | 369.83 | 44.097 | 3 | Alkane | Propane |
| 3.796 | 425.12 | 58.12 | 4 | Alkane | Butane |
| 3.03 | 507.5 | 86.18 | 6 | Alkane | Hexane |
| 2.74 | 540.1 | 100.2 | 7 | Alkane | Heptane |
| 2.49 | 568.7 | 114.23 | 8 | Alkane | Octane |
| 2.57 | 543.9 | 114.23 | 8 | Alkane | 2,2,4-Trimethylpentane |
| 2.11 | 617.7 | 142.28 | 10 | Alkane | Decane |
| 1.82 | 658.8 | 170.33 | 12 | Alkane | Dodecane |
| 1.4 | 722.2 | 226.44 | 16 | Alkane | Hexadecane |
| 1.07 | 768 | 282.5 | 20 | Alkane | Eicosane |
| 0.7272 | 828.3 | 394.8 | 28 | Alkane | Octacosane |
| 0.47 | 872 | 507 | 36 | Alkane | Hexatriacontane |
| 0.3627 | 910.37 | 647.2 | 46 | Alkane | Hexatetracontane |
| 5.041 | 282.34 | 28.054 | 2 | Alkene | Ethene |
| 3.143 | 504 | 84.161 | 6 | Alkene | 1-Hexene |
| 2.92 | 537.3 | 98.188 | 7 | Alkene | 1-Heptene |
| 2.68 | 567 | 112.21 | 8 | Alkene | 1-Octene |
| 4.895 | 562.05 | 78.11 | 6 | Aromatic | Benzene |
| 4.108 | 591.75 | 92.14 | 7 | Aromatic | Toluene |
| 3.61 | 617.1 | 106.16 | 8 | Aromatic | Ethylbenzene |
| 3.54 | 617 | 106.16 | 8 | Aromatic | m-Xylene |
| 3.209 | 631 | 120.19 | 9 | Aromatic | Cumene |
| 3.232 | 649.1 | 120.19 | 9 | Aromatic | 1,2,4-Trimethylbenzene |
| 2.71 | 760 | 168.238 | 13 | Aromatic | Diphenylmethane |
| 4.073 | 553.58 | 84.16 | 6 | Cycloalkane | Cyclohexane |
| 3.471 | 572.19 | 98.19 | 7 | Cycloalkane | Methylcyclohexane |
| 4.05 | 748.4 | 128.17 | 10 | Polycyclic aromatic | Naphthalene |
| 3.65 | 720 | 132.2 | 10 | Polycyclic aromatic | 1,2,3,4-Tetrahydronaphthalene |
| 2.87 | 869 | 178.23 | 14 | Polycyclic aromatic | Phenanthrene |
| 2.61 | 938.2 | 202.25 | 16 | Polycyclic aromatic | Pyrene |
| 0.7 | 822 | 422.8 | 30 | Terpene | Squalane |

**Table S2.** Frequently used transfer functions.

| Transfer function | Mathematical expression |
| --- | --- |
| tansig or tanh | $f\left( x \right)= \frac{e^{x}-e^{-x}}{e^{x}+e^{-x}}=\frac{2}{1-e^{-2x}}-1$ |
| logsig or sigmoid | $f\left( x \right)=\frac{1}{1+e^{-x}}$ |
| Sinusid | $f\left( x \right)=sin(x)$ |
| Purelin | $f\left( x \right)=x$ |
| Arctan | $f\left( x \right)={tan}^{-1}(x)$ |
| Rectified Linear Unit (ReLU) | $f\left( x \right)=max(0,x)$ |

**Table S3.** EOSs Formulas used in this work.

| Reference | PVT relation | EOS |
| --- | --- | --- |
| ^1^ | $P=\frac{RT}{\nu-b}-\frac{\alpha}{T^{1/2}\nu(\nu+b)}$ | ZJ: Zudkevitch-Joffe |
| ^2^ | $P=\frac{RT}{\nu-b}-\frac{\alpha}{\sqrt{T}\nu(\nu+b)}$ | RK: Redlich-Kwong |
| ^3^ | $P=\frac{RT}{\nu-b}-\frac{a_{c}\alpha}{\nu(\nu+b)}$ | SRK: Soave-Redlich-Kwong |
| ^3^ | $P=\frac{RT}{\nu-b}-\frac{\alpha}{\nu(\nu+b)+b\left(\nu-b\right)}$ | PR: Peng-Robinson |
| ^4,5^ | $\tilde{a}=\frac{A}{kTN}=\tilde{a}^{hc}+\tilde{a}^{id}+\tilde{a}^{disp}+\tilde{a}^{assoc}$ | Perturbed-Chain Statistical Associating Fluid Theory (PC-SAFT) |

**Table S4.** EOSs parameters and mixing rules.

| Reference | Parameters | EOS |
| --- | --- | --- |
| ^1^ | Parameter *α and b* are calculated as functions of temperature and pressure. For complex mixtures, $b_{i}=b_{i}^{ZJ}\left[1+b_{0}\left(\frac{T}{T_{C}}-1\right)\right]$  | ZJ: Zudkevitch-Joffe |
| ^2^ | $\begin{matrix} a=0.42748\frac{R^{2}T_{C}^{2.5}}{P_{C}} \\ b=0.08664\frac{RT_{C}}{P_{C}} \end{matrix}$ | RK: Redlich-Kwong |
| ^3^ | $\begin{matrix} a_{C}=0.42747\frac{R^{2}T_{C}^{2}}{P_{C}} \\ b=0.08664\frac{RT_{C}}{P_{C}} \\ m=0.480+1.574\omega-0.176\omega^{2} \\ \alpha=\left[1+m\left(1-\sqrt{T_{r}}\right)\right]^{2} \end{matrix}$ | SRK: Soave-Redlich-Kwong |
| ^3^ | $\begin{matrix} \alpha=a_{C}\alpha(T) \\ \alpha(T)=\left[1+m\left(1-\sqrt{T_{r}}\right)\right]^{2} \\ a_{C}=0.45724\frac{(RT_{C})^{2}}{P_{C}} \\ m=0.37464+1.54226\omega-0.26992\omega^{2} \\ b=0.07780\frac{RT_{C}}{P_{C}} \end{matrix}$ | PR: Peng-Robinson |
| ^2,6^ | $\begin{matrix} a=\sum_{i=1}^{N} \sum_{j=1}^{N} z_{i}z_{j}\sqrt{a_{i}a_{j}}\left[1-k_{ij}(T)\right] \\ b=\sum_{i=1}^{N} z_{i}b_{i} \end{matrix}$ | Van der Waals one-fluid mixing rules |
| ^4,5^ | $\begin{matrix} \tilde{\text{a}}^{\text{hc}}=\bar{m}\tilde{\text{a}}^{\text{hs}}+\tilde{\text{a}}^{\text{chain}}=\bar{m}\tilde{\text{a}}^{\text{hs}}-\sum_{i} x_{i}(m_{i}-1)\ln g_{ij}^{hs} \\ \bar{m}=\sum_{i} x_{i}m_{i} \\ \tilde{\text{a}}^{\text{hs}}=\frac{1}{\zeta_{0}}\left[\frac{3\zeta_{1}\zeta_{2}}{1-\zeta_{3}}+\frac{3\zeta_{2}^{3}}{\zeta_{3}(1-\zeta_{3})^{2}}+\left(\frac{\zeta_{2}^{3}}{\zeta_{3}^{2}}-\zeta_{0}\right)\ln(1-\zeta_{3})\right] \\ \zeta_{n}=\frac{\pi}{6}\rho\sum_{i} x_{i}m_{i}d_{i}^{n}\text{ }n\in\left\{0,1,2,3\right\},\text{ }\eta=\zeta_{3} \\ d_{i}=\sigma_{i}\left[1-0.12\exp\left(-3\frac{\varepsilon_{i}}{kT}\right)\right] \\ g_{ij}^{hs}=\frac{1}{1-\zeta_{3}}+\left(\frac{d_{i}d_{j}}{d_{i}+d_{j}}\right)\frac{2\zeta_{2}}{(1-\zeta_{3})^{2}}+\left(\frac{d_{i}d_{j}}{d_{i}+d_{j}}\right)^{2}\frac{2\zeta_{2}^{2}}{(1-\zeta_{3})^{2}} \\ \tilde{\text{a}}^{\text{dis}}=-2\pi\rho I_{1}\left(\eta,\bar{m}\right)\overline{m^{2}\varepsilon\sigma^{3}}-\pi\rho\bar{m}C_{1}\left(\eta,\bar{m}\right)I_{2}\left(\eta,\bar{m}\right)\overline{m^{2}\varepsilon^{2}\sigma^{3}} \\ I_{1}\left(\eta,\bar{m}\right)=\sum_{i=0}^{6} a_{i}(\bar{m})\eta^{i}\text{ , }I_{2}\left(\eta,\bar{m}\right)=\sum_{i=0}^{6} b_{i}(\bar{m})\eta^{i} \end{matrix}$  where *a_i_* and b_i_ depend on the chain length as given in Gross and Sadowski ^4^. $\begin{matrix} C_{1}=\left[1+\bar{m}\frac{8\eta-2\eta^{2}}{\left(1-\eta\right)^{4}}+(1-\bar{m})\frac{20\eta-27\eta^{2}+12\eta^{3}-2\eta^{4}}{\left[\left(1-\eta\right)\left(2-\eta\right)\right]^{2}}\right] \\ \overline{m^{2}\varepsilon\sigma^{3}}=\sum_{i} \sum_{j} x_{i}x_{j}m_{i}m_{j}\left(\frac{\varepsilon_{ij}}{kT}\right)\sigma_{ij}^{3} \\ \overline{m^{2}\varepsilon^{2}\sigma^{3}}=\sum_{i} \sum_{j} x_{i}x_{j}m_{i}m_{j}\left(\frac{\varepsilon_{ij}}{kT}\right)^{2}\sigma_{ij}^{3} \\ \varepsilon_{ij}=\sqrt{\varepsilon_{i}\varepsilon_{j}}\left(1-k_{ij}\right) \\ \sigma_{ij}=\frac{\left(\sigma_{i}+\sigma_{j}\right)}{2} \end{matrix}$   The expressions for the contributions from the dispersion and ideal gas are identical to those of Gross and Sadowski ^4^. | Perturbed-Chain Statistical Associating Fluid Theory (PC-SAFT) |

**Table S5.** Pure-component PC-SAFT EOS parameters for the substances used in this work.

| Reference | *ε/k* (K) | *σ* (Å) | *m* | *M_w_* (g/mol) | Substance |
| --- | --- | --- | --- | --- | --- |
| ^7^ | 33.85 | 4.244 | 0.4874 | 2.016 | Hydrogen |
| ^4^ | 242.78 | 3.8373 | 3.8176 | 114.231 | Octane |
| ^4^ | 243.87 | 3.8384 | 4.6627 | 142.285 | Decane |
| ^4^ | 287.35 | 3.6478 | 2.4653 | 78.114 | Benzene |
| ^8^ | 255.62 | 3.9789 | 11.083 | 394.77 | Octacosane |

**Table S6.** The optimized values of the binary interaction parameter for the EOSs in different solubility systems.

| Reference | *k_ij_* | Temperature (K) | EOS | System |
| --- | --- | --- | --- | --- |
| ^9^ | 0.27 | 298.15 | PR | Hydrogen + Octane |
|  | 0.31 | 323.15 |  |  |
|  | 0.33 | 373.15 |  |  |
|  | 0.29 | 298.15 | SRK |  |
|  | 0.34 | 323.15 |  |  |
|  | 0.37 | 373.15 |  |  |
| This work ^*^ | 0.71 | 298.15 | RK |  |
|  | 0.74 | 323.15 |  |  |
|  | 0.85 | 373.15 |  |  |
|  | -0.12 | 298.15 | ZJ |  |
|  | -0.16 | 323.15 |  |  |
|  | -0.19 | 373.15 |  |  |
|  | -0.06 | 298.15 | PC-SAFT |  |
|  | -0.06 | 323.15 |  |  |
|  | 0.00 | 373.15 |  |  |
| ^10^ | 0.34 | 432 | PR | Hydrogen + Decane |
|  | 0.34 |  | SRK |  |
| This work ^*^ | 0.35 |  | RK |  |
|  | -0.21 |  | ZJ |  |
|  | -0.04 |  | PC-SAFT |  |
| ^10^ | 0.29 | 303.15 | PR | Hydrogen + Benzene |
|  | 0.29 |  | SRK |  |
| This work ^*^ | 0.32 |  | RK |  |
|  | -0.15 |  | ZJ |  |
|  | -0.08 |  | PC-SAFT |  |
| ^11^ | 0.1697 | 423.2 | PR | Hydrogen + Octacosane |
|  | 0.1697 |  | SRK |  |
| This work ^*^ | 0.917 |  | RK |  |
|  | -0.78 |  | ZJ |  |
|  | -0.05 |  | PC-SAFT |  |

^*^The binary interaction parameter has been regressed from the experimental solubility data.

**References**

1 Ronze, D., Fongarland, P., Pitault, I. & Forissier, M. Hydrogen solubility in straight run gasoil. *Chemical Engineering Science* **57**, 547-553 (2002).

2 Pedersen, K. S., Christensen, P. L. & Shaikh, J. A. *Phase behavior of petroleum reservoir fluids*. (CRC press, 2014).

3 Nasrifar, K., Bolland, O. & Moshfeghian, M. Predicting natural gas dew points from 15 equations of state. *Energy & fuels* **19**, 561-572 (2005).

4 Gross, J. & Sadowski, G. Perturbed-chain SAFT: An equation of state based on a perturbation theory for chain molecules. *Industrial & engineering chemistry research* **40**, 1244-1260 (2001).

5 Chen, Y., Mutelet, F. & Jaubert, J.-N. l. Modeling the solubility of carbon dioxide in imidazolium-based ionic liquids with the PC-SAFT equation of state. *The Journal of Physical Chemistry B* **116**, 14375-14388 (2012).

6 Kwak, T. & Mansoori, G. Van der Waals mixing rules for cubic equations of state. Applications for supercritical fluid extraction modelling. *Chemical engineering science* **41**, 1303-1309 (1986).

7 Florusse, L., Peters, C., Pamies, J., Vega, L. F. & Meijer, H. Solubility of hydrogen in heavy n‐alkanes: Experiments and saft modeling. *AIChE journal* **49**, 3260-3269 (2003).

8 Tihic, A., Kontogeorgis, G. M., von Solms, N. & Michelsen, M. L. Applications of the simplified perturbed-chain SAFT equation of state using an extended parameter table. *Fluid phase equilibria* **248**, 29-43 (2006).

9 Valderrama, J. O. & Cisternas, L. A. Binary interaction parameters in cubic equations of state for hydrogen—hydrocarbon mixtures. *Chemical engineering science* **45**, 49-54 (1990).

10 Qian, J.-W., Jaubert, J.-N. & Privat, R. Phase equilibria in hydrogen-containing binary systems modeled with the Peng–Robinson equation of state and temperature-dependent binary interaction parameters calculated through a group-contribution method. *The Journal of Supercritical Fluids* **75**, 58-71 (2013).

11 Park, J., Robinson, R. L. J. & Gasem, K. A. Solubilities of hydrogen in heavy normal paraffins at temperatures from 323.2 to 423.2 K and pressures to 17.4 MPa. *Journal of Chemical and Engineering Data* **40**, 241-244 (1995).
